# A Model of DNA Repeat-Assembled Mitotic Chromosomal Skeleton

**DOI:** 10.3390/genes2040661

**Published:** 2011-09-26

**Authors:** Shao-Jun Tang

**Affiliations:** Department of Neuroscience and Cell Biology, University of Texas Medical Branch, Galveston, TX 77555-1069, USA; E-Mail: shtang@utmb.edu; Tel.: +1-409-772-1190

**Keywords:** repetitive DNA, DNA repeats, mitotic chromosome, chromosomal skeleton, repeat pairing, chromatin

## Abstract

Despite intensive investigation for decades, the principle of higher-order organization of mitotic chromosomes is unclear. Here, I describe a novel model that emphasizes a critical role of interactions of homologous DNA repeats (repetitive elements; repetitive sequences) in mitotic chromosome architecture. According to the model, DNA repeats are assembled, via repeat interactions (pairing), into compact core structures that govern the arrangement of chromatins in mitotic chromosomes. Tandem repeat assemblies form a chromosomal axis to coordinate chromatins in the longitudinal dimension, while dispersed repeat assemblies form chromosomal nodes around the axis to organize chromatins in the halo. The chromosomal axis and nodes constitute a firm skeleton on which non-skeletal chromatins can be anchored, folded, and supercoiled.

## 1. Introduction

Packaging of lengthy chromatins into mitotic chromosomes is a key step for eukaryotic cells to faithfully transmit genetic information during mitosis. The reproducible characteristics of chromosomal karyotypes and banding indicate that the daunting task of the packaging is achieved by precisely controlled chromatin folding processes. In spite of intensive investigation for decades and the many models proposed, the organizational principle of chromatin fibers (10 or 30-nm fibers of the DNA-histone complex) in packaged mitotic chromosomes remains elusive [1,2]. In an influential model, chromatins are folded into radial loops that are attached to a longitudinal axis formed by non-histone ‘scaffold’ proteins [3–6]. However, the *in vivo* role of such a contiguous proteinaceous axis in chromosomal condensation is controversial [1,7–9]. Another model suggests chromosomal packaging by hierarchical folding (see [10] and refs therein), yet the underlying mechanism is unclear. Based on internal biophysical properties, a recently proposed model views mitotic chromosomes as chromatin networks with crosslinking in approximately 10- to 15-kb intervals [8,11]. The molecular nature of the crosslinkers, however, is not known. These and other models [12–14] have emphasized a central role for trans-acting proteins in organizing mitotic chromosomes.

Here, a radically different model is presented that highlights an organizer activity for DNA repeats in specifying the architecture of mitotic chromosomes. This structure was devised with a basic assumption of repeat pairing (RP), a tendency of interactions or associations of homologous DNA repeats in the cell, and with the constraints suggested by the published data on the spatial organization of repetitive DNAs in mitotic chromosomes. Somatic pairing of homologous chromatins is a well-established universal phenomenon playing a critical role in polytene chromosome formation, recombination and transvection. Association of linearly interspersed homologous repetitive DNAs in spatial proximity, which strongly suggests RP, has been documented in many experimental observations [15–23]. Extensive evidence of RP and its potential roles in chromatin organization was discussed in details in another paper [24]. The potential mechanisms by which homologous repeats pair (or interact) were also postulated [24]. The model presented here focuses on the central idea that DNA repeats function as chromatin organizer modules to guide fiber folding and crosslinking in mitotic chromosomes. An important feature of the model is that the architecture of mitotic chromosomes is organized by a DNA repeat-assembled skeleton.

## 2. Outline of the Model

**Figure 1:**
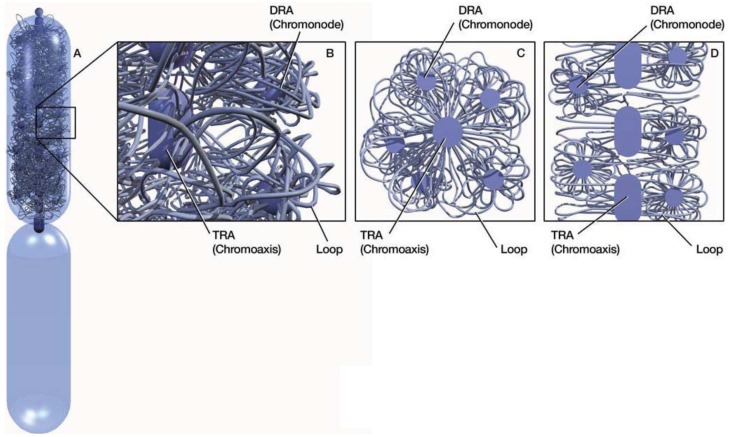
Proposed chromatin organization in mitotic chromosomes. (**A**) A model of a metaphase chromosome. Chromatin organization is only depicted for the top half of the chromosome. A chromoaxis, consisting of a central column of tandem repeat assemblies (TRAs) (rounded segments), and chromonodes, consisting of dispersed repeat assemblies (DRAs) (spheres around the chromoaxis), are buried in non-skeletal chromatin mass (strings). (**B**) An enlarged portion of the metaphase chromosome. (**C**) A cross-section of the metaphase chromosome perpendicular to the chromoaxis. (**D**) A longitudinal section of the metaphase chromosome along the chromoaxis. The diagrams are highly simplified and are meant to illustrate the principle rather than structural details of chromatin organization within the chromosomes. TRAs and DRAs, displayed as solid structures, are densely packaged chromosomal domains resulting from repeat pairing (RP). Non-skeletal chromatins are folded into various loops that are further packaged into supercoils, which, for simplicity of illustration, are not depicted in the diagrams (see Fig. 2). Different loops may be crosslinked via RPs (not shown).

In mitotic chromosomes, DNA repeats in the same family interact or pair with one another to form compact repeat assemblies (RAs). The RAs collectively constitute a chromosomal skeleton (chromoskeleton) on which non-skeletal chromatins are anchored (Figure 1). A chromoskeleton consists of two types of RA-based structures with specific chromatin organizing functions: the chromosomal axis (chromoaxis) and chromosomal node (chromonode). The chromoaxis consists of a centrally aligned array of tandem repeat assemblies (TRAs) and acts as the axial organizer that specifies the longitudinal and rod morphology of a mitotic chromosome. Via RP, the chromoaxis ‘attracts’ chromatin regions with homology to the axial tandem repeats to form radial loops. Chromonodes are dispersed repeat assemblies (DRAs) in the halo. Chromonodes fold the radial loops into smaller loops for supercoiling. Next, I shall summarize the main features and relevant supporting evidence for the model.

## 3. Chromoaxis

A major structural element in the chromoskeleton model is the chromoaxis, which is a tandem array of TRAs on the longitudinal axis (Figure 1). The chromoaxis and TRAs in mitotic chromosomes are analogous to the spine and vertebrae in the human body, respectively. The proposed segmental TRA organization of chromoaxes is consistent with the idea of modular axes [12]. Although current data suggest that tandem repeats are the major components of chromoaxes, it is unclear if all tandem repeats are organized into chromoaxes.

The existence of a TRA-formed chromoaxis is suggested by the fact that tandem repeats are often condensed to form axial structures of metaphase chromosomes, such as centromeres and telomeres. The alignment of tandem repeat blocks along the axis in other chromosomal regions has been shown by fluorescent *in situ* hybridization (FISH) experiments [25]. Because satellite tandem repeats are often AT-rich [26], the hypothesis of TRA-constituted chromoaxes predicts that there is a central axis that consists of segmented and condensed TA-rich DNA. This prediction is generally consistent with the staining patterns of mitotic chromosomes as revealed by DPAI, which preferably binds AT-rich sequences (Figure 3 in [27]). In addition, previous studies clearly revealed AT-queues on chromatid axes [28].

**Figure 2:**
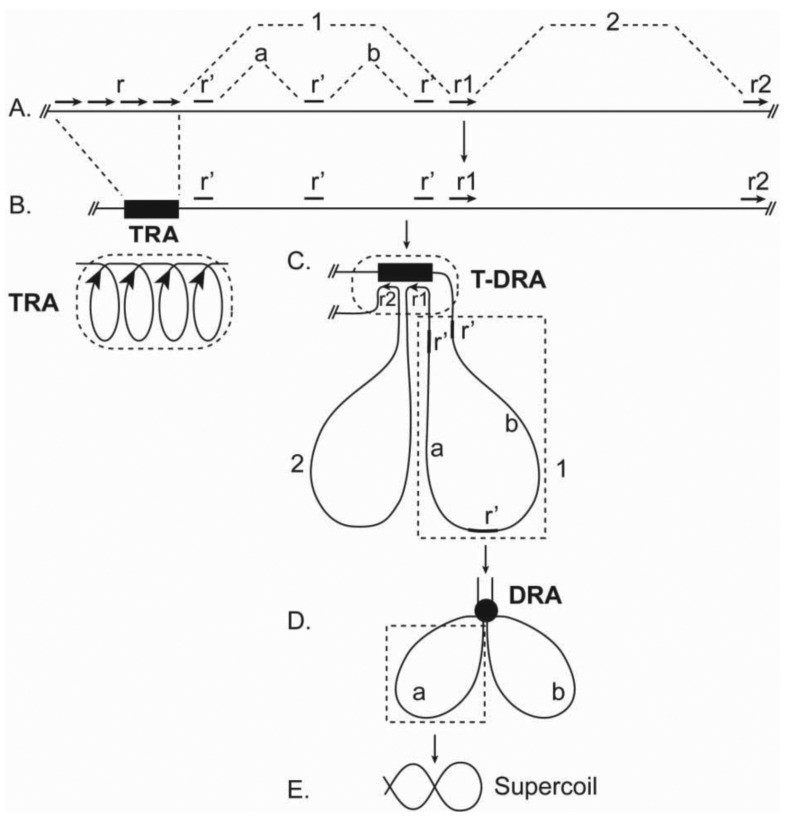
Hypothetic mechanisms of chromatin folding and chromoskeleton formation in mitotic chromosomes. (**A**) A chromatin segment containing tandem repeats (r), dispersed repeats (r′), and repeats homologous to tandem repeats (r1, r2). Intervals between repeats are indicated by 1, 2, a, and b. (**B**) Repeat pairing (RP) among tandem repeats results in the formation of tandem repeat assembly (TRA), which is represented by a black rectangle. A more detailed coiled structure of TRA in which adjacent repeats are paired is depicted.(**C**) RP of r1, r2, and TRA causes the formation of a T-DRA and the transformation of chromatin segments 1 and 2 into primary (radial) loops. (**D**) RP of dispersed r's in primary loop 1 leads to the formation of a DRA and secondary loops a and b. (**E**) Closed loops (loop a) can be further packaged by supercoiling. Shown here is chromatin folding on only one TRA (one chromoaxial segment); multiple such structural units can juxtapose to form a whole mitotic chromosome with a chromoaxis, DRAs (chromonodes), and chromatin loops around the axis.

TRAs are proposed to be compact structures in which adjacent tandem repeats are paired in a coiled chromatin structure (Figure 2A, B). This hypothesis predicts that the chromoaxis is an axial structure with AT-rich coils. Coincidently, AT-coils were observed on the central axis of mitotic chromosomes [28], although it is not yet known if the observed AT-coils correspond directly to the postulated TRA coils or a higher-order structure containing the latter.

If the postulated chromoaxis is indeed a tandem array of TRA coils, such an axial coil would be an ideal structural foundation for the longitudinal and rod morphology of metaphase chromosomes. Therefore, disruption of TRA coils would cause mitotic chromosomes to lose these gross morphological features. Indeed, incubation of the AT-hook protein MATH-20, which preferentially binds AT-rich satellite sequences, transformed longitudinal and rod mitotic chromosomes into spherical structures. MATH-20 likely interfered with interactions of AT-queues, thus disrupting the TRA chromoaxis [29].

How would chromoaxes organize chromatins in mitotic chromosomes? Because of the expected high density of tandem repeats in TRAs, chromoaxes may act as a ‘pairing sink’ for dispersed homologous sequences in non-chromoaxial chromatins. This type of RPs causes the formation of tandem-dispersed repeat assemblies (T-DRAs) and chromatin loops radiating from the chromoaxis (Figure 2C). This model then predicts the existence of dispersed *cis*-elements that are homologous to tandem repeats and play critical roles in radial loop formation. Indeed, previous work identified scaffold/matrix attachment regions (SARs; MARs) as the cis-elements that interact with the ‘scaffold’ and define the base of the radiating loops [30,31]. Importantly, SARs are homologous to satellite tandem repeats [31–33]. Thus, SARs may mediate radial loop formation by pairing with TRAs. Observations of radial loops have been well documented [4,34].

A non-histone ‘scaffold’ was proposed as the organizing axis of mitotic chromosomes [5]. TOPO II and condensins, the major components of ‘scaffolds’ are known to bind to AT-rich sequences (SARs and satellite tandem repeats) and structured or knotted DNA, respectively [28,35–37]. These DNA binding properties indicate that chromoaxes are suitable binding substrates for these ‘scaffold’ proteins. The chromoskeleton model predicts that these ‘scaffold’ proteins should, at least partially, co-localize with TA-rich coils (*i.e.*, TRAs) and follow a spiral distribution pattern on the axis. These predictions are consistent with published observations [6,28]. Thus, ‘scaffold’ proteins may form complexes with the TRA chromoaxis and modulate the latter. Under this framework, ‘scaffold’ proteins may contribute to chromosome organization by interacting with TRAs.

## 4. Chromonodes

Another critical chromoskeletal element in the postulated model is the DRA-formed chromonodes that are distributed around the chromoaxis and organize halo chromatins (Figure 1). The idea of DRA chromonodes in the halo is consistent with the observations of spatial clustering of dispersed repeats in mitotic chromosomes. For example, pea (*Pisum sativum L.*) retrotransposon dispersed repeats Psat32 and Psat3-26 visualized by FISH were confined in globular domains in metaphase chromosomes [18]. Similar spatial patterns were observed for other disperse repeats [16,38–40]. Because dispersed repeats in the same family are individually distributed in the linear genomes in general [Some transposons can nest with others in the same or different families. However, as indicated by a recent study [41], only a small percentage of transposons are expected to nest with members in the same family.], their globular, rather than diffused, FISH signals in mitotic chromosomes were consistent with the idea of spatial clustering. This clustering was presumably a result of RPs. Because of the harsh experimental conditions, less stable DRAs may have been disassembled after FISH).

If the hypothesis of chromonode formation by dispersed repeat clustering is correct, during mitotic chromosome packaging, a transition of the spatial organization of specific dispersed repeats from a more distributive pattern in a relatively relax chromosome to a more clustered pattern in a compact chromosome is expected. This prediction is consistent with observed FISH signals of dispersed repeats in maize pachytene and metaphase chromosomes [(Tekay in Figures 1 and 2) in ref [39]]. Such a phenomenon may only be observable for repeats whose DRAs are stable enough to maintain after FISH.

Here, it is proposed that DRA chromonodes are halo organizers (Figure 1). A major mechanism for chromonodes to organize halo chromatins is likely folding radial loops into smaller loops by RPs among dispersed repeats in the same radial loop (Figure 2D). Consequently, the formation of chromonodes further packages chromatins. Dispersed repeats on different chromatin loops may also pair. This type of RP would cause loop crosslinking to form an integrative chromatin network in the halo.

Notably, proposed chromonodes and their emitting loops (Figure 1) resemble the observed rosette structures released from chromosome preparations [42].

## 5. Chromatin Loops and Supercoils

The third important element of the model is chromatin loops. They are the chromosomal ‘flesh’ attached on the chromoskeleton.

In this model, chromatins in the halo are folded into hierarchical levels of loops (Figure 2). (i) Primary loops. RP between TRAs and their homologous sequences, such as SARs, leads to the formation of primary loops, radiating from the chromoaxis (Figure 2C). (ii) Secondary loops. Further folding of primary loops, due to RP among dispersed repeats in the same primary loop, forms secondary loops (Figure 2D). It is possible that the proposed primary and secondary loops correspond to the observed loops radiating from axial and rosette structures, respectively [4,34,42]. In theory, secondary loops can be folded further by RPs.

Because loops in this model are closed circular chromatin structures, they may be folded further by supercoiling, which can dramatically reduce loop volumes and is an important mode of chromosomal packaging [43]. Scaffold proteins such as condensins, which preferably associate with knots of interconnected chromatins, have helicase activity, and are implicated in mitotic chromosomal packaging [35,44], may bind to the loop base on DRAs and facilitate supercoiling (Figure 2E).

## 6. Cautions

Despite a critical role of the DNA-repeat-based chromoskeleton emphasized in this model, the potential involvement of trans-acting factors such as ‘scaffold’ proteins in mitotic chromosome organization is not downplayed. As indicated earlier, these proteins may interact with the chromoskeleton and modulate the stability of the latter. The structure described here is compatible with many published data, and the fairly detailed specifics of the model can be directly tested.

This model suggests that packaging of mitotic chromosomes follows reproducible pathways specified by RPs. However, these pathways should not be viewed as rigid. Instead, because one repeat may have multiple potential pairing partners, a certain degree of structural plasticity of mitotic chromosomes is expected, especially at a micro-level. Elucidating these repeat-governed morphogenetic pathways will be essential for fully understanding the molecular basis of mitotic chromosomal organization.

According to this model, the chromoskeleton is a core structure that is buried in mitotic chromosomes and is sensitive to cell state changes. These properties may create technical difficulties for its characterization.

## 7. Conclusions

A model for chromatin organization in mitotic chromosomes is proposed. According to this model, DNA repeats assemble via RP into core chromoskeletal structures, including chromoaxes and chromonodes, to coordinate the packaging of chromatins. Chromoaxes are the longitudinal organizers, while chromonodes are the halo organizers. These skeletal structures govern the architecture and morphology of mitotic chromosomes. Non-skeletal chromatins anchor to the chromoskeleton as loops, which are further packaged by supercoiling. RP among the different loops may generate a large integrative chromatin network within the halo.

Previous studies on the molecular mechanism of mitotic chromosome organization have mainly focused on identifying a protein core with an organizing activity. *In vitro* experiments indicated that mitotic cell extracts could condense naked DNAs from different sources [45]. However, despite important discoveries of modulatory roles of specific proteins, such as condensins and TOPO II, a protein that is absolutely required for mitotic chromosome packaging has not been identified so far. The novel perspective suggested in this paper points to a different direction for the mechanistic pursuit. Under this new paradigm, DNA repeats are the key internal organizer modules in mitotic chromosomes. Their coordinated interactions provide a driving force to generate chromatin order during mitotic chromosomal condensation. By creating repeats within genomes, evolution may have stumbled on a ‘simple’ solution for eukaryotic cells' intimidating task of packaging the long genetic fiber for faithful transmission.
